# Draft Genome Sequence of the White-Rot Fungus Phanerochaete sordida YK-624

**DOI:** 10.1128/MRA.00842-21

**Published:** 2021-10-21

**Authors:** Toshio Mori, Hideo Dohra, Tomohiro Suzuki, Hirokazu Kawagishi, Hirofumi Hirai

**Affiliations:** a Faculty of Agriculture, Shizuoka University, Shizuoka, Japan; b Research Institute of Green Science and Technology, Shizuoka University, Shizuoka, Japan; c Graduate School of Science and Technology, Shizuoka University, Shizuoka, Japan; d Center for Bioscience Research and Education, Utsunomiya University, Utsunomiya, Japan; Vanderbilt University

## Abstract

Here, we report the draft genome sequence of the white-rot basidiomycete fungus Phanerochaete sordida YK-624 (ATCC 90872), which was isolated in Yakushima, Kagoshima, Japan. The genome of this strain was found to be 41.2 Mbp, with a G+C content of 58.7%, and to comprise 17,108 predicted protein-coding sequences.

## ANNOUNCEMENT

Phanerochaete sordida YK-624 is a white-rot basidiomycete fungus that exhibits superior ligninolytic properties (1). The fungus was isolated from decaying wood collected in Yakushima, Japan. A piece cut from the decaying wood xylem was placed on wood agar medium (0.2% beech wood meal, 0.02% guaiacol, and 1.6% agar [pH 5.5]). Grown hyphae were isolated after incubation at 30°C for 7 days. The fungus secretes manganese peroxidase as the primary ligninolytic enzyme during wood decay, in addition to lignin peroxidases (2). P. sordida YK-624 also degrades several recalcitrant organic pollutants, such as neonicotinoid insecticides (3–5), endocrine-disrupting compounds (6, 7), and polychlorinated dioxins (8). It has been suggested that cytochrome P450s (CYPs) are involved in many of these reactions (3–5, 7). In addition, a molecular breeding technique for the fungus has been established, and several transformants have been constructed (e.g., references 2, 9, and 10). Therefore, the fungus can be applied as a platform for the analysis of white-rot fungus-specific gene function and biotechnological applications. However, basic genetic information regarding P. sordida, such as genes encoding lignin-degradation-related enzymes and CYPs, remains unknown. To enhance understanding of the metabolic mechanism and to facilitate modification of the activities of this fungus, we sequenced the genome of P. sordida YK624.

Phanerochaete sordida YK-624 (ATCC 90872) was pregrown on potato-dextrose agar (PDA) plates. Two mycelium-covered plugs (1.0-cm diameter) from the PDA plates were inoculated into 100-ml Erlenmeyer flasks containing 10 ml of potato-dextrose liquid medium. The cultures were incubated for 5 days at 30°C, and then mycelia were recovered. Genomic DNA was extracted using the Isoplant II kit (Nippon Gene Co., Ltd.), followed by ethanol precipitation for final purification. The DNA concentration was determined using a Quantus fluorometer (Promega, USA). DNA fragmentation was performed using an M220 focused ultrasonicator (Covaris Inc., USA), and libraries were prepared using a TruSeq DNA PCR-free library preparation kit (Illumina, USA). Whole-genome sequencing using a MiSeq system (Illumina) was carried out using 301-bp paired-end reads, resulting in a total of 36,391,668 paired-end reads (totaling 10,038 Mb). Raw sequence data were cleaned using Trimmomatic v. 0.38 by trimming the adaptor sequences and bases with quality scores of <15 and filtering out reads longer than 150 bp (11). The resultant 22,452,994 high-quality reads, totaling 5,521 Mb, were assembled using SPAdes v. 3.13.0 with a default *k*-mer set and the options careful, only-assembler, and cov-cutoff auto (12). The assembled draft genome sequence of P. sordida consisted of 341 contigs (>2,000 bp), with a total genome size of 41.2 Mb, an *N*_50_ value of 414,756 bp, and an average G+C content of 58.7%. Protein-coding genes were predicted using BRAKER2 v. 2.1.0 with the fungus option (13) and then were manually curated. The P. sordida YK-624 genome contained 17,108 protein-coding genes, and their completeness, as analyzed using BUSCO v. 3.0.2 with the polyporales_odb10 data set, reached 4,409/4,464 markers (98.7%) (14). Peroxidase genes, including 8 manganese peroxidases, 8 lignin peroxidases, a DyP-type peroxidase, and more than 200 putative CYP genes, were annotated. In addition, some laccase-like (multicopper oxidase) genes were also found, although laccase proteins have not been identified in P. sordida YK-624. These data suggest that P. sordida YK-624 probably degrades a variety of recalcitrant organic compounds, such as lignin, using these enzymes.

### Data availability.

The draft genome sequence of P. sordida YK-624 (ATCC 90872) was deposited in DDBJ/ENA/GenBank under accession number BPQB01000000. The raw read sequences were deposited in the DDBJ under the accession numbers DRR311115 and DRR311116.
